# Development of a Taste-Masked Orodispersible Film Containing Dimenhydrinate

**DOI:** 10.3390/pharmaceutics4040551

**Published:** 2012-10-26

**Authors:** Maren Preis, Miriam Pein, Jörg Breitkreutz

**Affiliations:** Institute of Pharmaceutics and Biopharmaceutics, University of Düsseldorf; Universitätsstraße 1, 40225 Düsseldorf, Germany

**Keywords:** Orodispersible film, orally disintegrating dosage form, taste-masking, electronic taste sensing, electronic tongues, dimenhydrinate, cyclodextrin, maltodextrin, solubility, solvent casting

## Abstract

Orodispersible dosage forms are promising new approaches for drug delivery. They enable an easy application, as there is no need to drink high amounts of liquids or swallow large solid dosage forms. The aim of the study was to develop an orodispersible film (ODF) as an alternative to tablets, syrups or suppositories for the treatment of vomiting and nausea, especially for the pediatric population. Formulations were investigated by X-ray diffraction, scanning electron and polarized light microscopy. Additionally, two commercially available electronic taste sensing systems were used to investigate the applied taste-masking strategies. Results obtained from X-ray-diffraction and polarized light microscopy showed no recrystallization of dimenhydrinate in the formulation when cyclodextrin or maltodextrin were used as solubilizing and complexing agent. All ODFs showed fast disintegration depending on the characterization method. In order to get taste information, the dimenhydrinate formulations were analytically compared to pure drug and drug-free formulations by electronic tongues. Results obtained from both systems are comparable and were used together for the first time. It was possible to develop an ODF of dimenhydrinate that is fast disintegrating even in small volumes of liquid. Furthermore, *in vitro* taste assessment by two electronic tongues revealed taste-masking effects by the excipients.

## 1. Introduction

Orally disintegrating dosage forms are promising new approaches to improve and simplify drug administration. Orodispersible formulations are beneficial especially for the pediatrics but also for the geriatric population as swallowing high volumes of liquids can be avoided [1]. Furthermore, risk of choking is minimized in oromucosal preparations, such as orodispersible films or buccal tablets and films, due to their possible adhesion to the oral mucosa or their fast disintegration [2].

**Figure 1:**
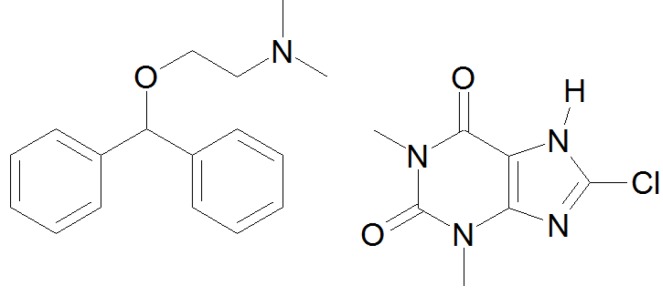
Chemical structure of dimenhydrinate (diphenhydramine + 8-chlorotheophylline).

The development of orodispersible films (ODF) containing dimenhydrinate (DMH) offers an alternative to conventional tablets, syrups and suppositories for the treatment of vomiting and nausea. Chemically, DMH is a salt of diphenhydramine and 8-chlorotheophylline (Figure 1). Diphenhydramine is an antihistamic drug that is antagonistic at the H1 receptor in order to prevent and treat nausea and motion sickness [3]. 8-chlorotheophylline is added to counteract drowsiness triggered by diphenhydramine. DMH is a so called over-the-counter (OTC) drug that is commonly used in self-medication. The science information of a marketed syrup containing DMH claims a single dose of 8.25 mg for children with a body weight of 6 kg-10 kg [4].

Since 2008 an expert committee of the World Health Organization (WHO) proposed a benefit of solid dosage forms in pediatric medicines, this study aims to develop a solid dosage form containing DMH suitable for children [5]. A single dose of 5 mg per film is appropriate, as it corresponds to a single dose of the aforementioned marketed syrup. Furthermore, this dose is even applicable in the treatment of younger or lightweight children. 

In this study different excipients were investigated which, in the first place, are known for their taste masking effects and, additionally, enhance the solubility of the poorly water-soluble DMH. Hence, recrystallization in the films may be prevented [6,7].

ODFs recently became part of the monograph “oromucosal preparations” of the European Pharmacopoeia. However, no requirements limiting disintegration time have until now been specified [2]. As they are supposed to disperse or disintegrate rapidly, disintegration of the film should correspond to complete drug release. In this study, disintegration within three minutes was defined as the appropriate limit, according to the monograph of orodispersible tablets [8].

Electronic taste sensing is gaining interest in formulation development, because poor taste is known to reduce therapy adherence in patients, particularly children. Therefore, commercially available electronic taste sensing systems (electronic tongues) should be used and obtained data should be processed with multivariate analyses [9]. These systems can be used to compare a new taste masking approach in formulations to a drug-loaded (poor taste is considered) and pleasant tasting drug-free preparation. Multivariate analysis, especially principal component analysis, of taste sensors offers an interesting way to illustrate taste masking capacities in a two-dimensional graphic.

Literature reveals, saliva flow of healthy children (mean age: 7.94 years) varies between 0.82-0.93 mL/min [10]. Supposing a saliva flow of 1 mL/min over three minutes and two milliliters of saliva already present in the oral cavity, an approximate and realistic volume of liquid in the mouth would be 5 mL. To avoid bias in taste information by dilution, an adapted sample preparation is therefore needed.

## 2. Materials and Methods

### 2.1. Materials

The materials used for ODF preparation are shown in Table 1.

**Table 1:** Materials and functions.

Substance	Function	Distributor	Brand name
dimenhydrinate	drug	Pharma Roth (D)	
modified pea starch polymer	film forming agent	Roquette (F)	Lycoat RS 720
glycerol (anhydrous)	plasticizer	Caesar & Loretz (D)	
water	solvent		
ethanol abs.	co-solvent	VWR (D)	
E 124 (red)	coloring agent	Caesar & Loretz (D)	
hydroxypropyl-β-cyclodextrin	solubilizer taste	Roquette (F)	Kleptose^®^ HPB oral grade
	masking agent		
maltodextrin (pea starch based)	solubilizer taste	Roquette (F)	Kleptose^®^ linecaps
	masking agent		
sulfobutylether-β-cyclodextrin (+sodium salts)	solubilizer taste	Cydex (US)	Captisol^®^
	masking agent		
saccharin sodium	sweetener	Caesar & Loretz (D)	

### 2.2. Sample Preparation

Cyclodextrin and maltodextrin formulations were premixed with dimenhydrinate in aqueous solution (1:1 molar ratio) and stirred for 24 h. Subsequently, polymer, plasticizer and coloring agent were added and films were prepared by solvent casting method: cyclodextrins, maltodextrin and saccharin sodium, respectively, were premixed with DMH in aqueous solution and stirred for 24 h until a clear solution was obtained. Polymer, plasticizer and coloring agent were added and solutions were stirred again. Solutions were poured onto a release liner that was fixed by vacuum suction on the film applicator (Erichsen film applicator, Erichsen, Hemer, Germany). Afterwards they were casted by the help of a coating knife (speed: 6 mm/s) at the calculated thickness to achieve desired drug amounts per film. Casting thickness *h* is calculated by inserting *m (Batch)*—the mass of the whole batch, *m (API p. film)*—the desired drug amount per film, *ρ (Batch)*—the density of the formulation, *m (API)*—the drug amount in the batch and *A (Film)*—the area of one film in Equation 1. A correction factor *f* of 130 µm was used due to coating knife adjustment. As actual values of film thickness showed a shift compared to the set values, shift behavior was defined beforehand over different coating thicknesses. Drug-free films were prepared accordingly and casted at the same thickness as the drug-loaded films. They were dried at room temperature for 24 h and cut into rectangular pieces (1.5 cm × 2 cm; drug content per film: 5 mg). Film thickness was determined by a micrometer screw (Mituyo, Neuss, Germany). Composition of films is shown in Table 2.


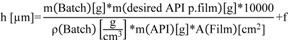
(1)

**Table 2:** Dimenhydrinate and drug-free formulations (x indicates that the particular ingredient is included in the formulation).

Batch code:	D	P	DCA	PCA	DCD	PCD	DCDS	PCDS	DMD	PMD	DS	PS
Dimenhydrinate	x	-	x	-	x	-	x	-	x	-	x	-
HP-β-CD	-	-	-	-	x	x	x	x	-	-	-	-
SBE-β-CD	-	-	x	x	-	-	-	-	-	-	-	-
Maltodextrin	-	-	-	-	-	-	-	-	x	x	-	-
Saccharin sodium	-	-	-	-	-	-	x	x	-	-	x	x

Film base: Lycoat RS 720; ethanol; distilled water; glycerol.

### 2.3. Film Thickness and Weight

Film thickness was determined by a micrometer screw (Mituyo, Neuss, Germany). Film weights were obtained by weighing single films on an analytical balance (Sartorius, Goettingen, Germany).

### 2.4. Drug Content

Dimenhydrinate content was determined by UV spectroscopy (Spekol 1200, Analytik Jena, Jena, Germany) at 277 nm. As recommended by the pea starch polymer’s supplier, films were completely dissolved in 100.0 mL of 0.1 M hydrochloric acid to ensure complete hydrolysis. Samples were diluted to reach a desired drug concentration of 2.5 mg/100 mL. Linearity was determined for DMH concentrations between 1 mg and 5 mg per 100 mL. Ten samples were measured per batch. Edge peaces with deviating thickness were excluded.

Content uniformity was determined by calculating acceptance values (AV) according to the European Pharmacopoeia 2.9.40. [10]. Quotient (*Q*) of actual content based on the arithmetic mean and target content was calculated.

### 2.5. Determination of Disintegration time

Methods were modified for the small sizes films of 1.5 × 1 cm^2^ from literature [12,13]. Method 1 (drop method): one film was placed onto a small glass beaker. One drop (0.2 mL) of distilled water was placed onto the film. Time until film break was measured. Method 2 (petri dish method): one piece of film was placed into a petri dish. After adding two milliliters of distilled water, the petri dish was shaken constantly. Time until the film fully disintegrated was measured.

### 2.6. Morphology

Crystallinty was investigated by X-ray diffraction (X’pert-MPD, Panalytical, Almedo, The Netherlands) and polarized light microscopy (Leica, Leica Microsystems Q500/550, Wetzlar, Germany). X’Pert-MPD was equipped with a Cu Kα point source (*λ* = 1.5406 Å). Measurement setup is shown in Table 3.

**Table 3:** Measurement setup for X-ray diffraction.

operational voltage	40 kV
operational amperage	40 mA
angular step size	0.0167113° 2 *θ*
range	10°-50° 2 *θ*
scanning rate	0.417782°/s

Gold sputtering was performed by Agar manual Sputter Coater B7340 (Agar scientific, Stansted, Essex, UK) and scanning electron microscopy (Leo 1430 VP, Leo Electron Microscopy, Cambridge, UK) was used for imaging.

### 2.7. Electronic Taste Sensing

Two commercially available systems were used: TS-5000Z (Insent, Atsugi-Chi, Japan) equipped with seven lipid membrane sensors corresponding to human taste attributes (3× bitter, salty, sour, umami and astringent) and α Astree (Alphamos, Toulouse, France) equipped with seven ChemFET-sensors for pharmaceutical use (ZZ, AB, BA, BB, CA, DA, JE), which are cross-selective (Table 4) [9]. An amount of 100 mL liquid sample was needed for electronic taste sensing, 20 ODFs were dissolved in 100.0 mL distilled water. This concentration corresponds to one dose in 5 mL, which is more suitable for taste assessments, as there is only a slight dilution of the samples. All samples were measured in triplicates. Measurements by Insent system were performed as recommended by the supplier. α Astree measurement setup was changed after validating different modes to improve repeatability [14]. The recommended measurement setup was changed from ABCABC to AABBCC (A, B, and C represent different sample concentrations). Sensors were dipped into a sample beaker and each sample was measured over a period of 120 s subsequently eight times. After this procedure, the sensors were dipped into a washing beaker, three times ten seconds, before the next sample was analyzed.

**Table 4:** Sensors of the electronic taste sensing systems.

Insent		α Astree	
SB2AAE	umami	ZZ	Cross selective
SB2CA0	sourness	AB	
SB2CT0	saltiness	BA	
SB2AE1	astringency	BB	
SB2AC0	bitterness	CA	
SB2AN0	bitterness	DA	
SB2C00	bitterness (anionic)	JE	

Principal component analysis was performed by multivariate statistic program Simca-P + V12 (Umetrics, Umea, Sweden). Sensor data was analyzed by the program in individual data sets. For data merging, sensor data from both systems was included in a mutual data set. Model was fitted by the system and principal components (PC) were calculated and displayed in two dimensional score scatter plots (PCA maps). PC loading plots were generated to show which type of values contribute to the formulations.

## 3. Results and Discussion

### 3.1. Film Properties

**Figure 2:**
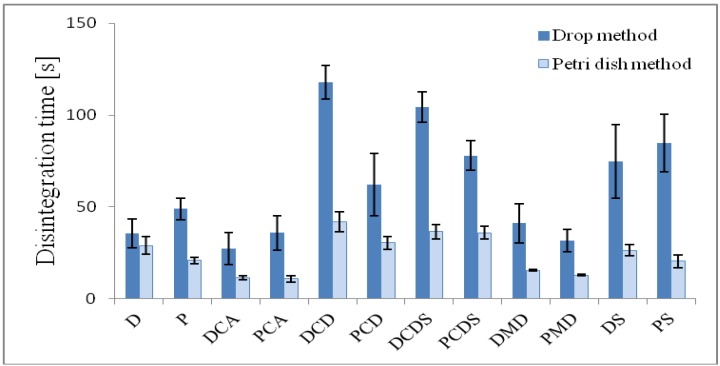
Disintegration times of prepared orodispersible films; *n* = 3; all values: arithmetic mean ± standard variation.

Properties of the developed ODFs are shown in Table 5. Mean disintegration times for all formulations varied between 10.87 s and 41.73 s for the petri dish method and between 27.4 s and 117.9 s for the drop method. Disintegration behavior varied depending on film thicknesses and weights. Thicker films (DCDS, DCD and DS) disintegrated slower. No excipient dependency could be concluded. Drop method resulted in longer disintegration times than those obtained by petri dish method (Figure 2). The petri dish method is more dynamic due to the slight shaking of the petri dish. Nevertheless, disintegration times of all films were in an acceptable range (<180 s) according to disintegration times of orodispersible tablets [8] specified in the European Pharmacopoeia. It has been observed that films became very sticky immediately after first water contact.

Investigated concentration series of DMH showed a linear response in UV measurements (coefficient of determination *R*^2^ = 0.998). Variation of drug content (AV = acceptance value) was satisfying for all formulations, even if the labeled amount of dimenhydrinate was not achieved in all formulations (*Q* [%] = 94.0−112.4). The low standard deviations led to the conclusion that the drug was homogeneously distributed in the formulations.

**Table 5:** Properties of prepared orodispersible films: thickness, weight, drug content and disintegration time.

	Thickness (µm)	Weight (µm)	Drug content			Disintegration time	
			(mg)	AV	*Q* (%)	Drop (s)	Petri dish (s)
D	143.4 ± 7.2	59.4 ± 2.3	4.7 ± 0.1	0.5	94.8	35.5 ± 7.8	29.1 ± 4.8
P	136.6 ± 7.0	55.6 ± 1.7	-	-	-	49.0 ± 5.9	21.0 ± 1.7
DCA	114.7 ± 3.2	50.5 ± 1.8	5.2 ± 0.1	0.3	104.5	27.4 ± 8.6	11.3 ± 1.1
PCA	116.0 ± 6.7	51.2 ± 3.3	-	-	-	36.0 ± 9.4	10.9 ± 1.7
DCD	158.0 ± 3.4	64.8 ± 1.1	4.7 ± 0.1	2.7	94.0	117.9 ± 9.3	41.7 ± 5.5
PCD	142.4 ± 25.4	66.8 ± 2.7	-	-	-	62.0 ± 17.0	30.6 ± 3.4
DCDS	171.2 ± 7.5	71.9 ± 3.0	5.6 ± 0.1	0.9	112.4	104.4 ± 8.2	36.6 ± 4.1
PCDS	152.7 ± 6.7	67.1 ± 5.2	-	-	-	78.1 ± 8.2	35.9 ± 3.4
DMD	125.0 ± 2.3	55.3 ± 2.6	5.0 ± 0.1	0.1	100.1	41.1 ± 10.6	15.5 ± 0.5
PMD	119.4 ± 9.4	52.2 ± 3.3	-	-	-	31.7 ± 6.0	12.9 ± 0.5
DS	156.6 ± 11.4	64.9 ± 3.9	5.5 ± 0.4	1.2	109.2	74.7 ± 20.1	26.4 ± 3.0
PS	127.5 ± 7.6	53.4 ± 2.7	-	-	-	85.0 ± 15.7	20.5 ± 3.6

All values: arithmetic mean ± standard variation: thickness, weight and drug content: *n* = 10; disintegration testing: *n* = 3.

### 3.2. Morphology

**Figure 3:**
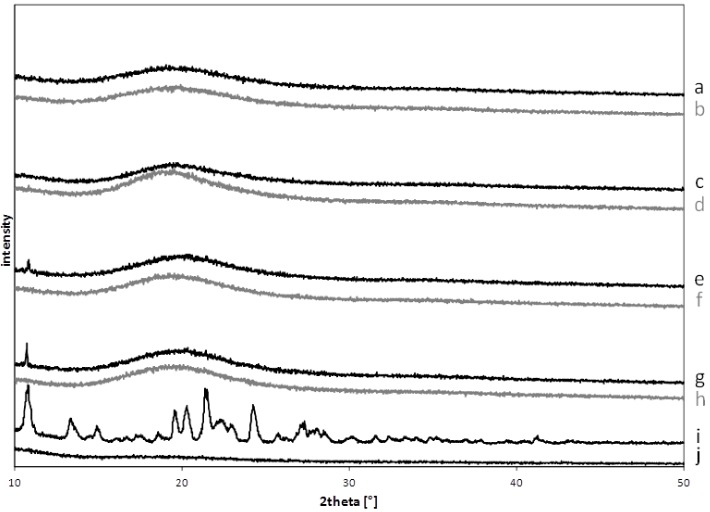
X-ray pattern of dimenhydrinate and drug-free formulations, pure dimenhydrinate and pure film forming polymer. (**a**) DMD; (**b**) PMD; (**c**) DCA; (**d**) PCA; (**e**) DS; (**f**) PS; (**g**) D; (**h**) P; (**i**) dimenhydrinate; (**j**)LycoatRS720(batch codes according to Table 2).

Crystallinity was only found for cyclodextrin and maltodextrin free films, respectively, indicating that drug solubility enhancement was given by these excipients not only during preparation in solution but also in solid state when films were dried and water was almost completely evaporated. Signal intensity was low, which can be explained by the low dose of the formulation and detection limits of the X-ray system (Figure 3).

**Figure 4:**
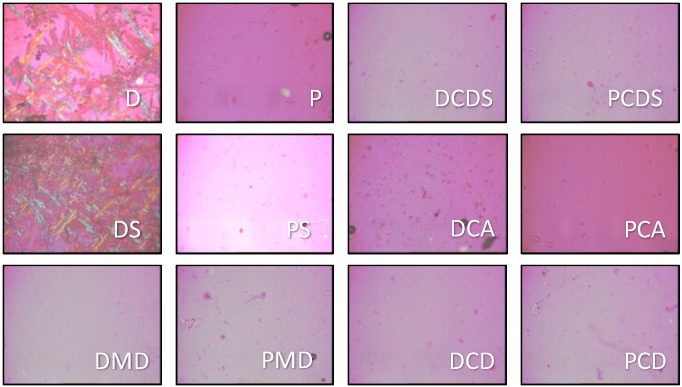
Polarized light microscopy: pictures of drug-free and drug-loaded films (batch codes according to Table 2).

**Figure 5:**
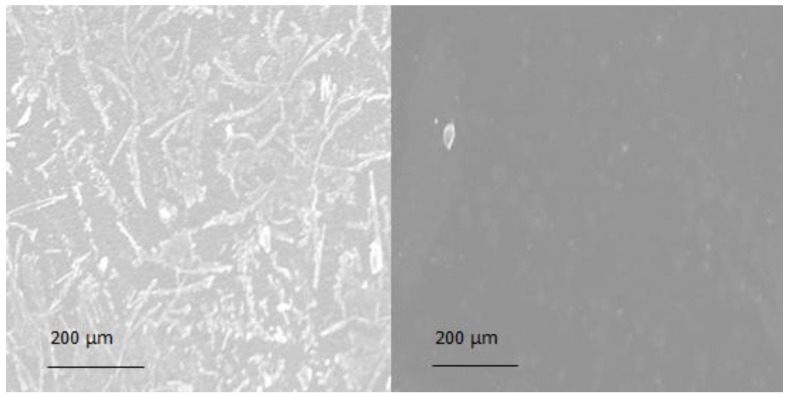
SEM surface images of drug loaded (**left**: D) and drug-free (**right**: P) formulation (batch codes according to Table 2).

After drying, films containing neither cyclodextrin nor maltodextrin appeared opaque, whereas all other formulations were transparent. Polarized light microscopy showed crystal growth in formulation D and DS (Figure 4). Surface images of a drug-loaded and a drug-free formulation obtained from scanning electron microscopy showed crystal growth on upper side (Figure 5).

### 3.3. Taste Assessment by Electronic Taste Sensing Systems

**Figure 6:**
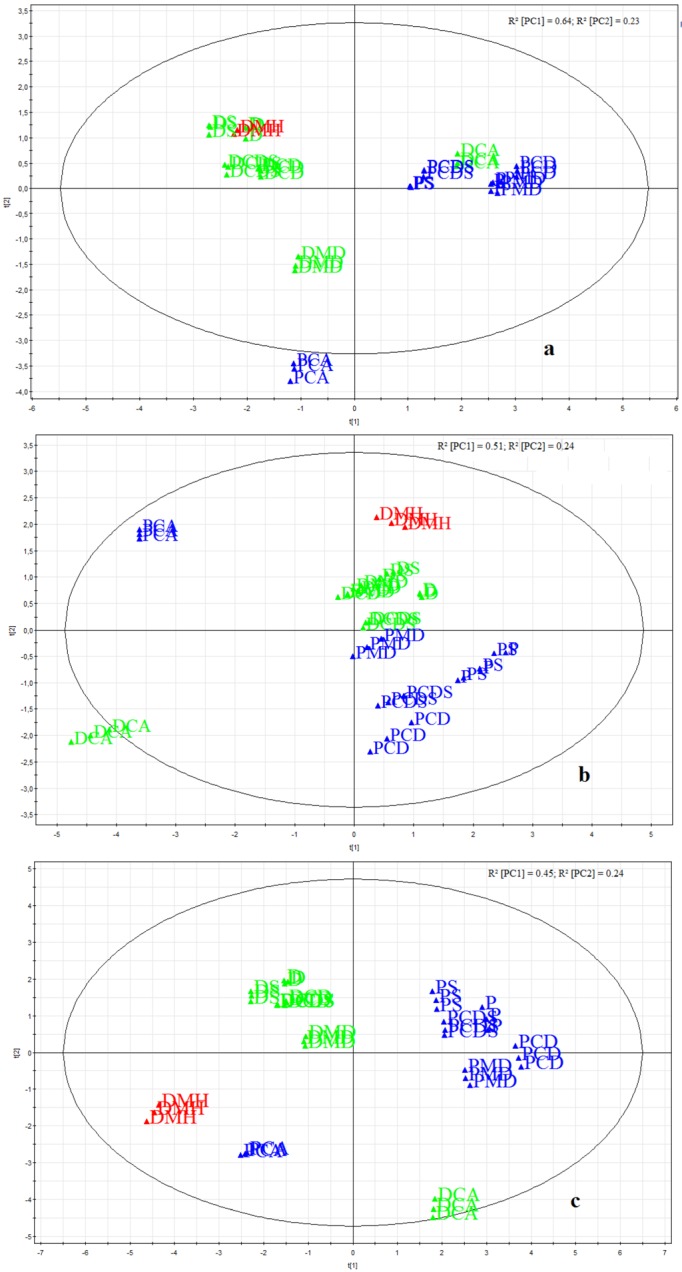
Principal component analysis: score scatter plots (**a**) Insent system; all seven sensors included;(**b**) αAstree system; all seven sensors included;(**c**) Insent and αAstree systems; all 14 sensors included.pure drug substance (red); drug-loaded formulations (green); drug-free formulations (blue).

**Figure 7:**
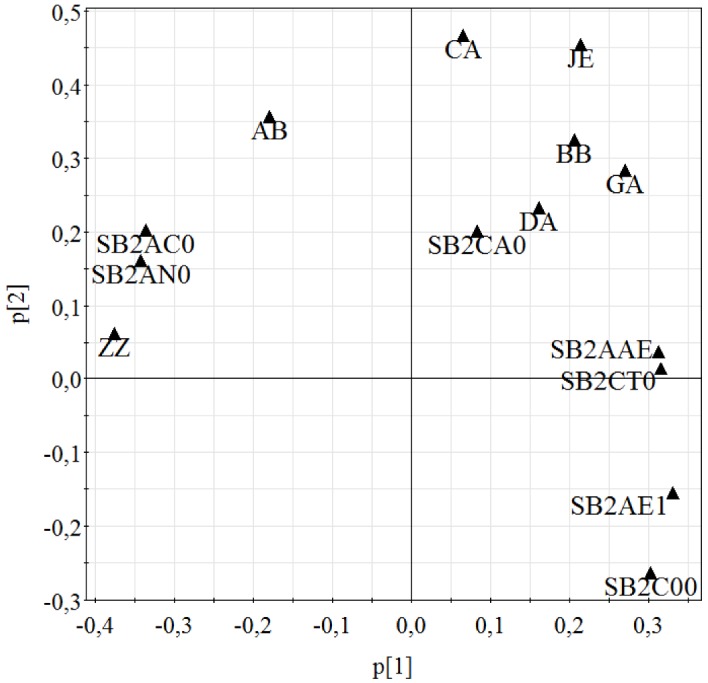
Loading plot of Figure 6c.

Comparative investigations of two different electronic taste sensing systems exhibited similar results in formulation testing. Both systems were able to distinguish between drug-free and drug-loaded formulations in principal component analysis PCA (Figure 6a,b). Captisol^®^ formulation (DCA) has been detected particularly by both systems. Insent system could not detect a difference between Captisol^®^ formulation (DCA) and drug-free formulations containing other added excipients (Figure 6a). Drug-free formulation containing Captisol^®^ (PCA) was detected completely different than other drug-free samples. As Captisol^®^ arrived as sodium salt, sensor responses differ from the other excipients. Thus, effects could be explained by different behavior of the more complex structure of the sulfobutylether-β-cyclodextrin. α Astree also detected differences between Captisol^®^ and HP-β-CD and maltodextrin formulations, respectively. Regarding the longest distance in Figure 5a and Figure 6b between pure drug and formulation, taste masking has been most successful for Captisol^® ^formulations. This result has been confirmed by both systems. A taste masking effect of the maltodextrin was also detectable by the Insent electronic tongue, whereas the αAstree system was able to distinguish between pure drug and non-taste masked formulations. Therefore, influences of the film forming polymer could be shown by α Astree electronic tongue only. Combining the sensor responses of both systems in multivariate data analysis showed improved discrimination between formulations, drug-free formulations and pure drug substance (Figure 6c). Combined data PCA-map Figure 6c revealed longest distance of DCA to pure drug substance compared to all other formulations. Drug-free Captisol^® ^formulation (PCA) was displayed closer to pure drug than drug-loaded DCA formulation, which can be explained by an occurring interaction between the cyclodextrin and DMH. For example, changes in molecular charge due to a complexation of DMH might cause the effect that is underlined in the loading plot of Figure 6c and Figure 7). DCA showed a shift towards Insent sensor SB2C00, which is sensible for charged components, especially anions.

## 4. Conclusions

Captisol^®^, HP-β-CD (Kleptose^®^ HPB oral grade) and maltodextrin (Kleptose^®^ linecaps 17) were able to improve the solubility of dimenhydrinate and could prevent the recrystallization of the drug substance in solid state of the film. Furthermore, solubility enhancers can be used as excipients for orodispersible films, not only because they ensure a uniform drug distribution in the film by avoiding irregular crystal growth, but also to improve the taste of these orodispersible formulations. 

An *in vitro* taste assessment by electronic taste sensing systems was successfully performed. As the drug is released in the oral cavity, a bad taste could worsen patient’s compliance especially when considering children. Hence, electronic tongues are able to distinguish between formulations, pure bad tasting drug and its non-taste-masked formulations. Therefore, successful taste masking can be assumed, when drug formulations are displayed close to drug-free and good tasting comparative formulations in PCA maps.

It was feasible to compare data from two electronic taste sensing systems but also to merge sensor responses, which even improved information on formulation and discrimination. Therefore, combining both systems could be a new promising tool in formulation development. This should be further tested for other drug substances. Already, the use of electronic taste sensing systems in orodispersible dosage form development is a new approach to confirm successful taste masking without the need of human taste panels in early stage of development.

To sum up, Captisol^®^ formulation was rated as the best formulation with respect to taste evaluation by both electronic taste sensing systems. Thus, it had the same advantages as the other cyclodextrin- and maltodextrin-based formulations, as the manufacturing resulted in homogenous ODFs showing no recrystallization of the drug compound. Regarding the maintenance of DMH in a non-crystallized state, maltodextrin Kleptose^®^ Linecaps was able to achieve the same effects in the ODFs as the cyclodextrins. Therefore, this maltodextrin with high amylose content offers an interesting alternative in ODF manufacturing with respect to pediatric formulations. Maltodextrins are already established in food industries, but also in supplementary feeding for babies and might be an uncritical additive to improve formulations.

ODFs in general represent a suitable dosage form for children, if the films are fast dissolving such as the presented ODFs and additionally have a pleasant taste. Due to the fact that the film forming polymer in this study became sticky in contact with minimal amounts of liquid, the risk of choking or inhaling parts of the dosage form is minimized. This low risk results even if the formulation needs one or two minutes to disintegrate completely, as it would adhere to any part of the oral cavity, e.g., the buccal or palatal site. 

Based on the acquired knowledge in this study, it can be concluded that ODFs are a promising dosage form. However, due to the novelty of the ODF monograph, pioneering work is necessary to ensure future high quality products on the European pharmaceutical market.

## References

[1] Hoffmann E.M., Breitenbach A., Breitkreutz J. (2011). Advances in orodispersible films for drug delivery. Expet. Opin. Drug. Deliv..

[2] European Pharmacopoeia Commission (2012). Oromucosal Preparations. European Pharmacopoeia 7.4.

[3] Watcha M.F., White P.F. (1992). Postoperative nausea and vomiting: Its etiology, treatment, and prevention. Anesthesiology.

[4] Astellas Pharma GmbH (2011). Vomex® A Sirup.

[5] World Health Orgnization (WHO) Report of the Informal Expert Meeting on Dosage Forms of Medicines for Children. 2008. http://www.who.int/selection_medicines/committees/expert/17/application/paediatric/Dosage_form_reportDEC2008.pdf.

[6] Loftsson T., Brewster M.E. (2010). Pharmaceutical applications of cyclodextrins: Basic science and product development. J. Pharm. Pharmacol..

[7] Ayenew Z., Puri V., Kumar L., Bansal A.K. (2009). Trends in pharmaceutical taste masking technologies: A patent review. Recent Pat. Drug Deliv. Formul..

[8] European Pharmacopoeia Commission (2008). Tablets. European Pharmacopoeia 7.0.

[9] Siqueira W.L., Nicolau J. (2002). Stimulated whole saliva components in children with Down syndrome. Spec. Care Dentist..

[10] European Pharmacopoeia Commission (2012). Uniformity of Dosage Forms (2.9.40.). European Pharmacopoeia 7.4.

[11] Garsuch V., Breitkreutz J. (2009). Novel analytical methods for the characterization of oral wafers. Eur. J. Pharm. Biopharm..

[12] Garsuch V., Breitkreutz J. (2010). Comparative investigations on different polymers for the preparation of fast-dissolving oral films. J. Pharm. Pharmacol.

[13] Woertz K., Tissen C., Kleinebudde P., Breitkreutz J. (2011). A comparative study on two electronic tongues for pharmaceutical formulation development. J. Pharmaceut. Biomed. Anal..

[14] Pein M., Eckert C., Preis M., Breitkreutz J. Taste sensing system αAstree as analytical tool—Performance Qualification using caffeine citrate as model substance. Proceedings of the 8th Pharmaceutics & Biopharmaceutics World Meeting.

